# Direct Potentiometric Study of Cationic and Nonionic Surfactants in Disinfectants and Personal Care Products by New Surfactant Sensor Based on 1,3-Dihexadecyl−1*H*-benzo[*d*]imidazol−3-ium

**DOI:** 10.3390/molecules26051366

**Published:** 2021-03-04

**Authors:** Nikola Sakač, Dean Marković, Bojan Šarkanj, Dubravka Madunić-Čačić, Krunoslav Hajdek, Božo Smoljan, Marija Jozanović

**Affiliations:** 1 Faculty of Geotechnical Engineering, University of Zagreb, 42000 Varaždin, Croatia; dubravka.madunic-cacic@saponia.hr; 2 Department of Biotechnology, University of Rijeka, 51000 Rijeka, Croatia; dean.markovic@biotech.uniri.hr; 3 Department of Food Technology, University North, 48000 Koprivnica, Croatia; bsarkanj@unin.hr; 4 Saponia Chemical, Pharmaceutical and Foodstuff Industry, Inc., 31000 Osijek, Croatia; 5 Department of Packaging, Recycling and Environmental Protection, University North, 48000 Koprivnica, Croatia; krunoslav.hajdek@unin.hr (K.H.); bozo.smoljan@unin.hr (B.S.); 6 Department of Chemistry, University of Osijek, 31000 Osijek, Croatia

**Keywords:** cationic surfactants, disinfectants, ionophore, nonionic surfactants, nose drops, personal care products, potentiometry, sensor

## Abstract

A novel, simple, low-cost, and user-friendly potentiometric surfactant sensor based on the new 1,3-dihexadecyl−1*H*-benzo[*d*]imidazol−3-ium-tetraphenylborate (DHBI–TPB) ion-pair for the detection of cationic surfactants in personal care products and disinfectants is presented here. The new cationic surfactant DHBI-Br was successfully synthesized and characterized by nuclear magnetic resonance (NMR), Fourier transform infrared (FTIR) spectrometry, liquid chromatography–mass spectrometry (LC–MS) and elemental analysis and was further employed for DHBI–TPB ion-pair preparation. The sensor gave excellent response characteristics for CTAB, CPC and Hyamine with a Nernstian slope (57.1 to 59.1 mV/decade) whereas the lowest limit of detection (LOD) value was measured for CTAB (0.3 × 10^−6^ M). The sensor exhibited a fast dynamic response to dodecyl sulfate (DDS) and TPB. High sensor performances stayed intact regardless of the employment of inorganic and organic cations and in a broad pH range (2−11). Titration of cationic and etoxylated (EO)-nonionic surfactant (NSs) (in Ba^2+^) mixtures with TPB revealed the first inflexion point for a cationic surfactant and the second for an EO-nonionic surfactant. The increased concentration of EO-nonionic surfactants and the number of EO groups had a negative influence on titration curves and signal change. The sensor was successfully applied for the quantification of technical-grade cationic surfactants and in 12 personal care products and disinfectants. The results showed good agreement with the measurements obtained by a commercial surfactant sensor and by a two-phase titration. A good recovery for the standard addition method (98–102%) was observed.

## 1. Introduction

Surfactants, or surface-active agents, lower the surface tension of water. A surfactant’s basic structure is a charged, hydrophilic head and a “fat” hydrophobic tail [1]. Based on the charge of the head, they are divided into anionic, cationic, amphoteric and nonionic categories. Surfactants are widely used as washing and cleaning agents. The market size for surfactants was US$39.901 million in 2019 and is projected to reach US$52.417 million by 2025, at a compound annual growth rate (CAGR) of 4.5% between 2020 and 2025 [2].

Cationic surfactant production remains at 6% of the global surfactant industry. They are used as disinfectants, bactericides, corrosion inhibitors and cleaning agents in home products and professional products in hospitals, industry, and agronomy as well as oilfield chemicals. The positively charged head of CSs allows them to stick to negatively charged surfaces, preventing electrostatic repulsion and act as textile softeners. Nonionic surfactants are amphipathic molecules that contain a lipophilic part, usually long alkyl chain or fatty acid, and a hydrophilic part such as an ethylene oxide chain of varying length, which means they are nonionizable in water [3]. Nonionic surfactants enhance the cleaning properties of cationic surfactants [4]. Formulations of cationic with nonionic surfactants are common in personal care products and pharmaceuticals [5,6].

Because of the global COVID-19 pandemic crisis, rising of world population, and the increasing industrialization of developing countries, the market demand for personal care products, disinfectants, detergents and cleaning agents is continuously growing [7]. The misuse and overuse of disinfectants as a result of heightened concerns regarding SARS-CoV−2 transmission through the skin or via environmental surfaces may have adverse effects on the skin or reproductive and respiratory systems [8]. Moreover, occupational exposure to quaternary ammonium compounds found in disinfectants are linked to increased risks associated with adverse respiratory conditions such as asthma, pulmonary cell damage and inflammation [9]. Consequently, in addition to many positive benefits, cationic surfactants may have a varied negative impact on human health and the environment [10,11]. Thus, new, fast, sensitive, and affordable methods of cationic surfactant determination in environmental samples and the quality control of commercial product formulations containing cationic surfactants are highly needed and have to be urgently developed. The regularly employed classical method for cationic surfactant determination is based on a two-phase titration with colorimetric signal change [12]. Even though the method has been recently improved [13], it has many drawbacks such as requiring the use of hazardous organic solvents or the need for experienced personnel and precise determination of errors for optical end-point readout.

Several new methods of detecting cationic surfactants based on luminescence [14], solid phase extraction–ion chromatography with conductivity detection [15], and fluorescence [16] have recently been established. However, because of complexity, increased price, or poor reproducibility, they are not suitable for routine analysis. On the other hand, taking into account their simplicity, speed, cost, reproducibility and usability as a routine analysis technique, ion-selective surfactant sensors for direct potentiometric titrations have been more and more considered as appropriate substitutions for the classical two-phase titrations and other methods.

Most surfactant sensors possess a liquid membrane-type sensor composed of an ionophore, a high molecular weight PVC, and a plasticizer [17,18]. The ionophore is the sensing material and is usually responsible for the selective interaction with the surfactant counter-ion and for the formation of the ion-pair. The ideal ionophore should have certain properties, including high stability and very low solubility, which further leads to the high sensitivity and increased changes of the potential in the inflexion point [18]. Therefore, the development of the new ionophores is of the high interest in order to enhance the electroanalytical properties of surfactant electrodes.

One of the possible major drawbacks of ion-selective surfactant electrodes could be the leaching of the sensor material that may result in their reduced lifetime [19]. The employment of low water-soluble hydrophobic ionophores can reduce leaching and enhance sensor properties. Recently, our group [20] and others [21] showed that the use of carbon-based nanomaterials in surfactant sensoring may lead to the significant enhancement of electrode properties as well as to a reduced cost of sensor fabrication. However, although the implementation of carbon nanotubes (CNTs) in electrochemical sensors has been shown to enhance some sensor functions, such as signal stability and lifetime, the strong interactions between inherently insoluble CNTs and polar solvents make these materials a challenge for the surfactant sensing [22]. Therefore, the development of new sensing materials and ionophores is highly desirable.

One ionophoric alternative is the preparation and use of new long-chain quaternary ammonium salts with high amphipathic character. It could improve sensor characteristics as well as ameliorate chemical interaction and recognition in ionophore binding sites [23], which depend on a sensitive balance between rigidity, required for selectivity, and flexibility, necessary for fast sensor response. The aromatic quaternary ammonium salts possess the required rigidity due to their aromatic rings as well as flexibility, because of free rotation around the exocyclic C–N bonds and within alkyl substituents.

The study of cationic surfactants in commercial product formulations where nonionic surfactants are present, represents an analytical challenge since nonionic surfactants have a negative influence on the direct potentiometric determination of cationic surfactants [24,25]. For this reason, it is important to investigate their influence on direct potentiometric titrations of cationic surfactants at different concentration levels and on different numbers of ethoxylated (EO) groups [26]. Apart from our group [25], several other studies developed direct potentiometric sensors for cationic surfactants [10,21,27], and some created mathematical models to predict the end-point break potential values [28].

The aim of this paper was to develop a novel direct potentiometric surfactant sensor based on the new 1,3-dihexadecyl−1*H*-benzo[*d*]imidazol−3-ium (DHBI) ionophore implemented as a DHBI–TPB sensing material in a PVC-based sensing membrane. Thus, the obtained novel DHBI–TPB surfactant sensor was fully characterized by analytical methods and further employed for the quantifications of cationic surfactants. The latter was performed by direct potentiometric titrations in model samples containing cationic and nonionic surfactants with different degrees of ethoxylation and in personal care products and disinfectants.

## 2. Results and Discussion

### 2.1. Synthesis and Characterization of the Quaternary Alkyl Ammonium Salt **2**

**Scheme 1:**
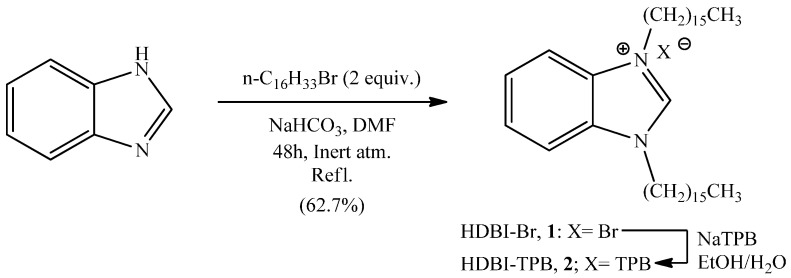
Synthesis of DHBI–TPB ion-pair sensing complex **2** via quaternary alkyl ammonium salt 1,3-dihexadecyl−1*H*-benzo[*d*]imidazol−3-ium bromide (**1**).

As shown in Scheme 1, quaternary alkyl ammonium salt DHBI-Br **1** was synthesized by the reaction of 1*H*−1,3-benzimidazole and 1-bromohexadecane. The reaction yield was established as 62.7%. The structure of the synthesized compound **1** was confirmed by IR, ^1^H- and ^13^C-NMR spectroscopy, mass spectrometry and elemental analysis (Supplementary Material, Figures S1–S4) established as 62.7%. The structure of synthesized compound 1 was confirmed by IR, 1H- and 13C-NMR spectroscopy, mass spectrometry and elemental analysis (Supplementary Material, Figures S1–S4).

### 2.2. Sensor Characteristics

After DHBI-Br **1** was synthesized, the counter ion exchange to obtain the ionophore **2** was performed by the controlled reaction of DHBI and a TPB (Scheme 1). The DHBI–TPB ionophore **2** was purified and incorporated into the PVC-based liquid-ion sensor membrane, which was then implemented into the Phillips ISE body electrode and used as a surfactant sensor for further investigations.

#### 2.2.1. Response

Response characteristics of the newly developed DHBI–TPB surfactant sensor were studied through the repetitive series of 5 cycles employing commercially available cationic surfactants regularly used in pharmaceutical and daily-care products as well as disinfectant formulations.

Generally, the direct potentiometric electromotive force of the cationic surfactant sensor with an ion-pair ionophore is based on the modified Nernstian equation:

(1) $$E=E_{0}+S\;log\;a_{SCat^{+}}$$

where E is the electromotive force of the surfactant sensor; $E_{0}$ is the constant potential term of the system; S is the slope of direct potentiometric surfactant sensor; $SCat^{+}$ is the activity of the positively charged surfactant ion.

The response of the DHBI–TPB surfactant sensor was studied for three typical cationic surfactants: hexadecyltrimethylammonium (CTAB), cetylpyridinium chloride (CPC) and Hyamine. Direct potentiometric response curves for each cationic surfactant were elaborated by MS Excel using a linear regression analysis to calculate the statistical data for the response characteristics of the DHBI–TPB surfactant sensor. Data were calculated for the 5-repetition cycle and slope values, intercept, correlation coefficient, limit of detection (LOD) and a useful concentration range for CTAB, CPC and Hyamine were measured, as shown in Table 1. The DHBI–TPB surfactant sensor showed excellent response characteristics for all three selected surfactants with a Nernstian slope; 59.2 mV/decade (CTAB), 58.4 mV/decade (CPC), 57.1 mV/decade (Hyamine), respectively. All three surfactants exhibited a high correlation coefficient in the effective linear concentration range. The broadest useful linear concentration range was achieved for CTAB, 2.0 × 10^−6^ to 1.0 × 10^−4^ M, and the lowest LOD value was acquired for same surfactant, 0.3 × 10^−6^ M, respectively. Both, CPC and Hyamine exhibited a useful linear concentration range from 3.0 × 10^−6^ to 1.0 × 10^−4^ M, slightly lower than for CTAB. In general, the DHBI–TPB surfactant sensor was exploited daily, and it exhibited an enhanced durability and signal stability over more than 4 months.

#### 2.2.2. Dynamic Response

**Figure 1:**
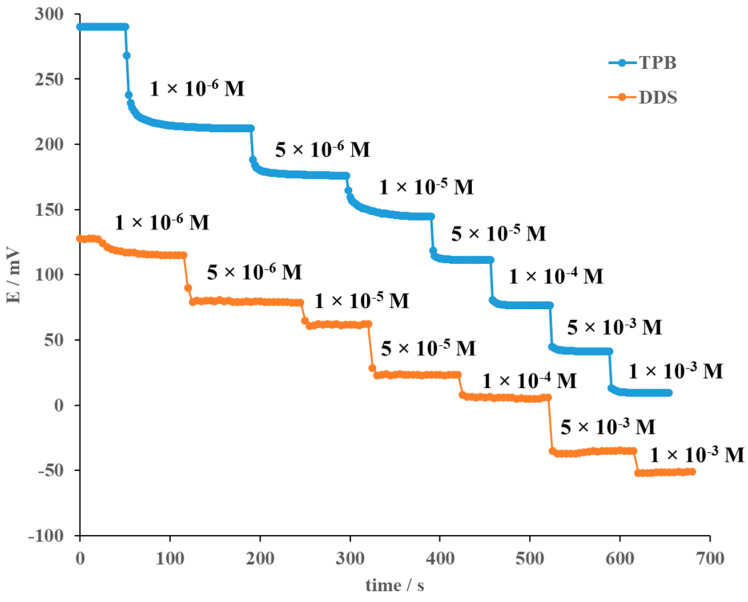
Dynamic response characteristics of the DHBI–TPB surfactant sensor towards TPB and DDS anions established by the addition of a determined concentration of selected anions.

Upon establishment of the response characteristic of the DHBI–TPB surfactant sensor towards cationic surfactants, a dynamic response on two selected anions––an organoboron–TPB and a dodecyl sulfate (DDS)––was measured. The dynamic response was evaluated by adding corresponding quantities of the TPB and DDS anions into deionized water in a broad concentration range from 1 × 10^−6^ to 1 × 10^−3^ M. The behavior of the DHBI–TPB surfactant sensor towards the affinity of TPB and DDS anions was observed (Figure 1) in terms of response time and signal stability. For both anions, the DHBI–TPB surfactant sensor had a fast response time (within few seconds) and fast signal stabilization even at low concentrations. The dynamic response study of the DHBI–TPB surfactant sensor towards the addition of TPB and DDS anions was used to predict the characteristics of direct potentiometric titration curves in terms of the change of sharpness of the signal and clarity of the inflexion point, which have a positive impact on sensor sensitivity and accuracy in end-point detection.

#### 2.2.3. Interferences

To characterize the DHBI–TPB surfactant sensor fully, an interference study was performed. Several inorganic and organic cations commonly present in commercial products formulations were used as potentially interfering ions. To verify the potential interferences, the CTAB cationic surfactant was selected since it gave the best response values and was cheaper than CPC. To calculate the selectivity coefficients for corresponding cations, the response of CTAB (10 µM to 10 mM) was measured in the interfering cation solution (20 mM). The influence of the interfering cation on the DHBI–TPB surfactant sensor response may be described by the Nikolskii-Eisenman equation. For the calculation of the selectivity coefficients ($K_{SCat_{determinated\;analyte\;ion}^{+}SCat_{interfering\;ion}^{+}}^{pot}$ or shorter $K_{SCat_{det.}^{+}SCat_{int.}^{+}}^{pot}$) a fixed interference method proposed by IUPAC [27] was used, and the calculated values are presented in Table 2. As shown, none of the investigated interferents had a significant influence on the DHBI–TPB surfactant sensor response towards CTAB.

#### 2.2.4. Influence of pH

**Figure 2:**
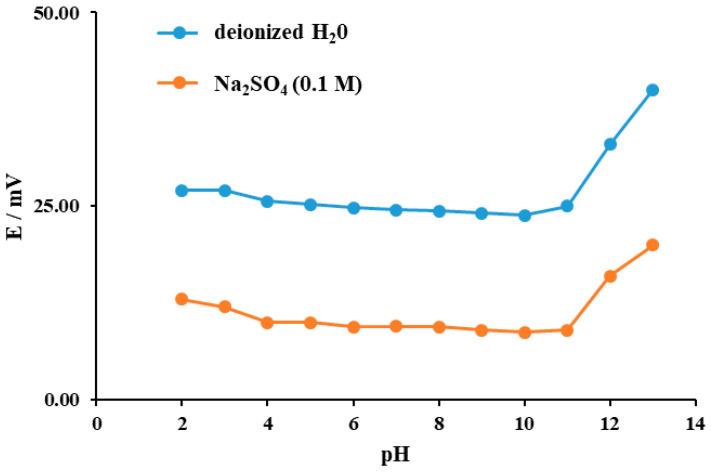
The study of the pH influence on the DHBI–TPB surfactant sensor response towards (1) hexadecyltrimethylammonium bromide (CTAB) (0.5 mM) in deionized water and (2) CTAB (0.5 mM) in Na_2_SO_4_ (0.1 M).

Since personal care products and disinfectants differ in their pH values, a study of the influence of pH on the DHBI–TPB surfactant sensor was carried out. The study was performed by the addition of the solutions of NaOH and HCl in the CTAB solution (0.5 mM) to adjust the pH from 2 to 13. To examine the effect of the increased ionic strength, the identical procedure was repeated with a mixture of CTAB (0.5 mM) in Na_2_SO_4_ (0.1 M). The potential readings of the potential stayed within ±1 mV. As presented in Figure 2, there is no significant influence of the pH on the response of the DHBI–TPB surfactant sensor within a broad pH range for both, low and high ionic strengths. The latter might be important because a single surfactant sensor could be used to test various commercial samples of different pH values with extraordinary simplicity and universality.

### 2.3. Potentiometric Titration

Since the DHBI–TPB surfactant sensor showed excellent response characteristics towards both TPB and DDS anions, direct potentiometric titrations of cationic surfactants were performed in two directions. For the first, the influence of nonionic surfactants and number of EO groups on direct potentiometric titrations, was examined by using TPB for the direct potentiometric titrations of CTAB and mixtures of CTAB with three different nonionic surfactants in the solution of Ba^2+^ salt. For the second, DDS was used for direct potentiometric titrations of commercial disinfectants and personal care products containing cationic surfactants, and the results obtained by the newly developed method with DHBI–TPB surfactant sensor were compared with the measurements obtained by the standard addition method.

#### 2.3.1. Titration of Model Samples Containing Cationic Surfactant and EO-Nonionic Surfactant

Disinfectants and personal care products often have formulations consisting of cationic and nonionic surfactants. Whereas the role of a cationic surfactant is to clean, disinfect or preserve, the major role of the nonionic surfactants is to provide better performance of the final formulations and because cationic surfactants are incompatible with anionic surfactants [4].

Direct potentiometric titrations of the cationic surfactant CTAB with the anionic titrant TPB can be described in two steps. First step is the formation of an ion pair:

(2) $$\mathrm{CTA}B^{+}+\mathrm{TP}B^{-}\underset{}{\Leftrightarrow }\mathrm{CTA}B^{+}\mathrm{TP}B^{-}$$

Barium ions (Ba^2+^), which are present in the solution, form a pseudoionic complex with EO groups from the nonionic surfactants

(3) $$Ba^{2+}+x\mathrm{EONS}\underset{}{\Leftrightarrow }{\left[Ba{\left(\mathrm{EONS}\right)}_{x}\right]}^{2+}$$

Thus, the reaction of EO-nonionic surfactant with TPB is possible according to:

(4) $${\left[Ba{\left(\mathrm{EONS}\right)}_{x}\right]}^{2+}+\;2\mathrm{TP}B^{-}\underset{}{\Leftrightarrow }Ba{\left(\mathrm{EONS}\right)}_{x}{\left(\mathrm{TPB}\right)}_{2}$$

**Figure 3:**
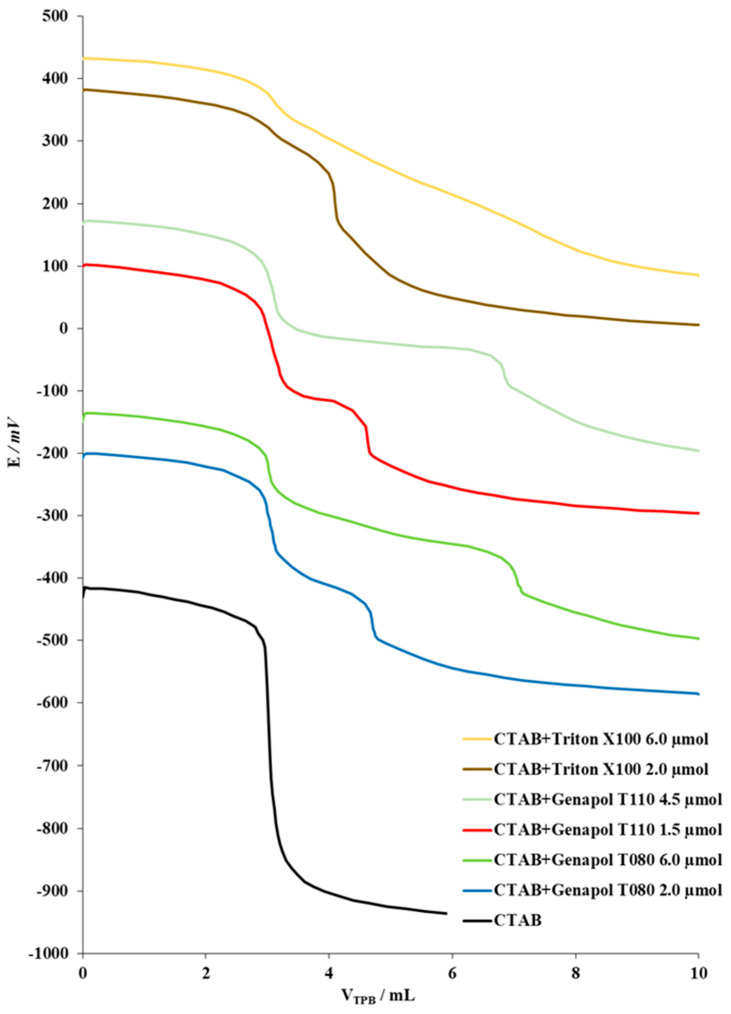
The influence of quantity and number of EO groups for the three EO-nonionic surfactants on the direct potentiometric titration curves of cationic surfactant CTAB (2.5 × 10^−3^ M) and selected EO-nonionic surfactants with TPB (2.5 × 10^−3^ M), in Ba^2+^ (0.2 M) using the DHBI–TPB surfactant sensor as the end-point detector. The titration curves for corresponding formulations of EO-nonioinic surfactant and CTAB were rearranged in order of appearance for clarity, from the bottom up: (1) only CTAB; (2) CTAB with Genapol T080 2.0 µmol; (3) CTAB with Genapol T080 6.0 µmol; (4) CTAB with Genapol T110 1.5 µmol; (5) CTAB with Genapol T110 4.5 µmol; (6) CTAB with Triton X100 2.0 µmol and (7) CTAB with Triton X100 6.0 µmol.

Since there is a difference between constants of the solubility of the products between the two ion pairs, the titration curves have two distinct inflexion points (Figure 3). Therefore, all titrations in this study were performed in the solutions containing Ba^2+^ ions (0.2 M) [3].

The solution of CTAB (2.5 × 10^−3^ M) was used as analyte and TPB (2.5 × 10^−3^ M) as a titrant. The direct potentiometric titration with the new DHBI–TPB surfactant sensor gave the titration curves a high potential change and a sharp inflexion point. Moreover, a series of three nonionic surfactants with different degrees of ethoxylation, namely, a different number of EO groups, were separately used in combination with CTAB as analyte and TPB as a titrant. The selected nonionic surfactants were Genapol T080 (2 µmol) with 8 EO groups, Genapol T110 (1.5 µmol) with 11 EO groups and Triton X100 (2 µmol) with 10 EO groups. Genapol surfactants were selected since they are often used in commercial product formulations and Triton X100 because it is used as a reference for determining EO-nonionic surfactants in effluents [29].

Figure 3 presents titrations of model samples of cationic surfactant CTAB (2.5 × 10^−3^ M) and three EO-nonionic surfactants that differ in quantity and number of EO groups with TPB (2.5 × 10^−3^ M) as the titrant obtained with the DHBI–TPB surfactant sensor as the end-point detector. As expected, all titration curves exhibited two inflexion points. The first inflexion was obtained because of the CTAB counter ion, and the second was due to the pseudoionic complex of Ba^2+^ ions and an EO-nonionic surfactant counter ion.

The direct potentiometric titration curves for the CTAB and for model solutions containing CTAB and EO-nonionic surfactants could be compared, and observations made of the extensive influence of the latter to the shape, the inflexion point at first equivalence point and the potential change. However, this effect was not so noticeable, especially when the number of EO groups was increased. The increment of EO groups also resulted in a lower change of the signal potential. The second inflection points were well defined for all EO-nonionic surfactants and were also well-matched with the volumes of the added EO-nonionic surfactants.

Furthermore, the concentration effect on three previously used EO-nonionic surfactants was examined as shown in Figure 3. The experiments were conducted with Genapol T080 (6.0 µmol), Genapol T110 (4.5 µmol) and Triton X100 (6.0 µmol). As shown, compared to the experiments conducted with the lower quantities of EO-nonionic surfactants, the effect of the increased concentration of the EOs number on titration curves was significant. Similar to the first inflexion points, the second were also less sharp, more stretched and with a lower change in potential; however, they were generally more disturbed. The smallest influence on the second inflexion point was observed for Triton X100 (6.0 µmol), as the inflexion was almost invisible.

The results confirmed the high influence of the concentration and number of EO groups of nonionic surfactants on cationic surfactants titrations. The higher concentrations of EO-nonionic surfactants were used, and a larger negative influence of the EO-nonionic surfactants was observed. Normally, EO-nonionic surfactant concentrations are less than double in commercial product formulations. Since the concentrations of EO-nonionic surfactants were tripled in the experiments, their expected influence on the shape and size of the cationic surfactant titration curves for the commercial products could be estimated as medium to low.

#### 2.3.2. Titration of Commercial Samples

Direct potentiometric titration of commercial samples using the DHBI–TPB surfactant sensor as the end-point detector was carried out in three directions. The first two were the direct potentiometric titration of a technical-grade cationic surfactant and the direct potentiometric titration of a cationic surfactant for the selected products. Both sets of results were compared with the results obtained by the standard addition method. The third concerned the direct potentiometric titration of 10 disinfectants and personal care products. The results were compared with commercial surfactant sensors. For all of these commercial samples, the pH values were adjusted to between 6 and 9.

The DHBI–TPB surfactant sensor was applied for direct potentiometric titrations of raw cationic surfactants that were used to produce disinfectants and personal care products. Thus, the experiments were performed with technical-grade CTAB, Hyamine, CPC, benzalkonium chloride (BAC), and didecyldimehtylammonium chloride (DDAC). The quantities of the cationic surfactants were evaluated from the end-point values (five repetitions) obtained by direct potentiometric titrations with DHBI–TPB surfactant sensor and TPB (4 × 10^−3^ M) used as a titrant (Table 3). The results showed that the purity of the employed technical-grade surfactants varied from 95.39 to 98.45%, while BAC and DDAC exhibited the lowest purity as expected. Finally, good agreement between the results obtained by direct potentiometric titration and the standard two-phase titration method [12] was obtained (Table 3).

Further studies of the influence of the matrix were performed by the direct potentiometric titration of a cation surfactant in commercial samples of three mouthwashes and eye drops using the DHBI–TPB surfactant sensor compared to the standard addition method (Table 4). As first, direct potentiometric titrations were employed to detect the quantity of cationic surfactants in commercial products. The DDS (4 × 10^−4^ M) was used as a titrant. The known quantity of CTAB (Table 4) was added to the samples and was titrated by a corresponding concentration of DDS. As presented, the recoveries were calculated from 98 to 102%.

**Figure 4:**
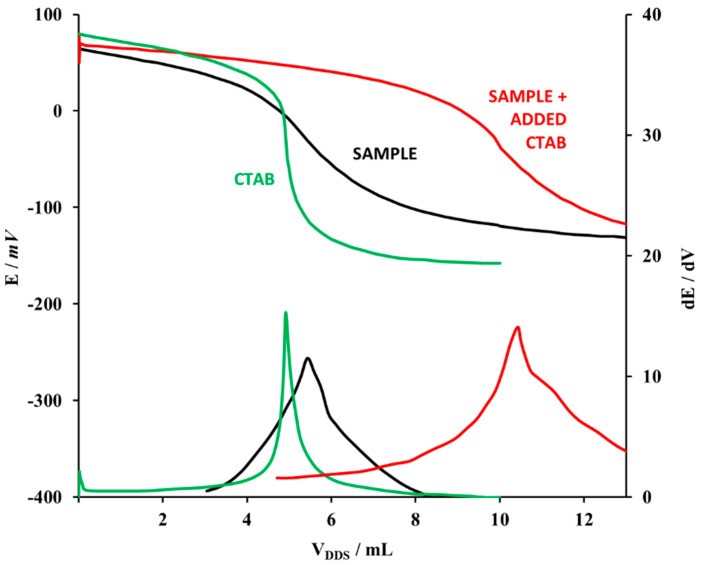
Direct potentiometric titration curves of (1) the commercial sample of the mouthwash 3 (black line); (2) CTAB (5 × 10^−4^ M) sample (green line); and (3) the same sample of mouthwash 3 with known addition of CTAB (red line); and their corresponding first derivatives. DDS (5 × 10^−4^ M) was used as the titrant.

In Figure 4 shows titration curves of the direct potentiometric titrations of cationic surfactants in commercial samples of mouthwash (Mouthwash 3) with and without added CTAB of known concentration and compares them with a titration of pure CTAB. DDS (5 × 10^−4^ M) was used as the titrant. The aim of Figure 4 is to compare the pure CTAB titration curve––high signal change, well-defined inflexion point, sharp and narrow first derivative, and clear end-point value (green line)––with the titration curve of the complex matrix in a commercial product containing a cationic surfactant––lower signal change, less-defined inflexion points and a broader first derivative curve, but still with a clear end-point value (black line).

After a known amount of CTAB was added to the sample, the end point was shifted for the corresponding equimolar volume of the DDS titrant. This proved that the sample matrix had no influence on the end-point detection, which was observed for all examined samples as presented in Table 4.

Twelve commercial disinfectants and personal care products were tested on cationic surfactants by direct potentiometric titration by using the DHBI–TPB surfactant sensor as the end-point indicator and TPB as a titrant as summarized in Table 5.

The pH values were adjusted between 3 and 9. The highest amounts of cationic surfactants were detected in both disinfectants for hospitals samples, 4.1032 and 5.0964%, respectively. In the mouthwash samples, cation surfactant amounts were much lower than in disinfectants for hospitals, ranging from 0.0480 to 0.1882%, respectively. In medical skin-disinfectant samples, cation surfactant amounts ranged from 0.1998 to 0.3769%. The lowest amounts of cationic surfactants were detected in all four nose drops samples where cation surfactant amounts found were in the range from 0.0099 to 0.0178%. The results showed an excellent agreement with the referent commercial surfactant sensor and a two-phase titration method results.

## 3. Materials and Methods

### 3.1. Reagents and Materials

Materials and reagents used for this organic synthesis were all analytical-grade chemicals: 1-bromohexadecane, with 1*H*−1,3-benzimidazole and NaHCO_3_ (Sigma Aldrich, Darmstadt, Germany). These chemicals were used in the experiments without any additional purification. Ultrapure water was used to prepare the solutions.

To conduct response characterizations and titrations, analytical-grade cationic surfactants were used: cetylpyridinium chloride (CPC) (Merck, Darmstadt, Germany); Hyamine 1622 (Hyamine) (Fluka, Buchs, Switzerland), hexadecyltrimethylammonium bromide (CTAB) (Fluka, Buchs, Switzerland) whereas cationic surfactants: benzalkonium chloride (BAC) and-didecyldimethylammonium chloride (DDAC) were technical grade. Anionic surfactant DDS (Fluka, Buchs, Switzerland) was used for the dynamic response and titrants were of an analytical grade. Nonionic surfactants, Genapol T 080 and Genapol T 110 were a technical-grade (Clariant, Switzerland) while Triton X−100 (Merck, Darmstadt, Germany) was analytical grade.

Na-TPB (Fluka, Buchs, Switzerland) was used for the response measurements and as a titrant for a pseudoionic complex with barium chloride (Fluka, Buchs, Switzerland).

For the membrane preparation, a high-molecular weight PVC (Sigma Aldrich, Darmstadt, Germany), a plasticizer o-nitrophenyloctylether (o-NPOE) (Sigma Aldrich, Darmstadt, Germany) and tetrahydrofuran (THF) (Merck, Darmstadt, Germany) analytical-grade chemicals were used.

### 3.2. Synthesis of Quaternary Alkyl Ammonium Salt 1

A novel quaternary alkyl ammonium salt DHBI-Br **1** was synthesized by reacting 1-bromohexadecane with 1*H*−1,3-benzimidazole. In a 25 mL round-bottomed flask equipped with a magnetic stir bar, 1*H*−1,3-benzimidazole (0.312 g, 2.64 mmol) and NaHCO_3_ (0.887 g, 10.56 mmol) in anhydrous dimethylformamide (15 mL) were dissolved. The resulting mixture was then stirred at 110 °C for 1 h in an inert atmosphere. After cooling the mixture to ambient temperature, an excess of 1-bromohexadecane (4.84 g, 15.84 mmol) was added, and the resulting mixture was again heated under reflux for 48 h. The progress of the reaction was followed by TLC (DCM/methanol = 10:0.25). The desired crude product 1 was obtained in high purity via filtration of the precipitated inorganic salts with methanol followed by filtration of the resulting waxy residue with hexane. This was followed by the flash column purification (DCM/methanol = 10:0.25) and drying under reduced pressure. The white powder bisalkylated DHBI-Br **1** (0.941 g, 1.66 mmol) was obtained in a 62.7% yield.

The structure of the synthesized DHBI-Br **1** was confirmed by IR, ^1^H- and ^13^C-NMR spectroscopy, mass spectrometry and elemental analysis. A detailed analysis can be found in the Supplementary Materials (Figures S1–S4).

### 3.3. Characterization of Quaternary Alkyl Ammonium Salt

#### 3.3.1. FTIR

IR spectra were recorded on a Shimadzu IR solution 1.30 FTIR−8400 S infrared spectrophotometer.

#### 3.3.2. NMR

^1^H NMR spectra were recorded at 600 MHz and ^13^C NMR spectra at 150.9 MHz using a Bruker AV600 spectrometer (Rheinstetten, Germany) at Ruđer Bošković Institute. Chemical shifts were referenced for the residual solvent peak (DMSO-d6) with SiMe_4_ as the internal standard.

#### 3.3.3. LC-MS

Spectroscopic information on the molecular ions was obtained through the API 2000 LC-ESI-MS/MS (Applied Biosystems, Foster City, CA, USA) in q1 ms scan mode.

#### 3.3.4. Elemental Analysis

For the elemental analysis PerkinElmer 2400 CHNS/O Series II System was used (PerkinElmer Inc., Waltham, MA, USA).

### 3.4. Preparation of DHBI–TPB Surfactant Sensor

#### 3.4.1. Preparation of DHBI–TPB 2 Ion-Pair Sensing Complex

For the ion-pair preparation, newly synthetized DHBI-Br **1** was used as a source of the cation and Na-TPB was used as a resource of a counter-ion. The mixture of an aqueous solution of Na-TPB (0.05 M) and ethanol/water solution (volume ratio 2:1) of DHBI-Br **1** was heated to 50 °C. The hot Na-TPB solution 10 mL was slowly added to the 30 mL of a hot DHBI-Br solution in ethanol-water containing equimolar quantity of DHBI-Br **1**. The opaque reaction mixture was stirred and heated. At 70 °C the mixture become transparent and the white precipitate appeared at 80 °C. After removing the white precipitate and evaporating the ethanol, the crude DHBI–TPB **2** salt was washed, filtrated, and dried at 80 °C. When the mass of the DHBI–TPB **2** was constant, it was stored in a desiccator and used for sensor membrane preparation.

#### 3.4.2. Sensor Membrane Preparation

The sensor membrane cocktail consisting of 33% high-molecular weight PVC, 66% plasticizer o-NPOE, and 1% prepared DHBI–TPB ion-pair **2** was prepared. The sensor membrane was made by the following procedure: 0.1 g of the above-described mixture was added to 2 mL of THF and sonicated for 15 min; the sensor membrane cocktail was slowly poured onto the borosilicate glass-ring mold fixed to the glass plate and left to dry. Upon drying, the glass ring was removed and the membrane was cut into smaller circular pieces to fit the electrode body.

#### 3.4.3. Surfactant Sensor Preparation

The cut sensor membrane was fixed to the Philips electrode body IS−561 (Supelco, Bellefonte, PA, USA) filled with NaCl (3 M) as an inner electrolyte. The obtained DHBI–TPB surfactant sensor was used for further studies.

### 3.5. Apparatus

Response, dynamic response, and interference measurements were carried out by Metrohm 794 Basic Titrino paired with a Metrohm 781 pH meter and corresponding stirrer. The system was controlled by home-developed software.

The titration measurements were carried out by Metrohm 808 Titrando equipped with an incorporated stirrer. The system was controlled by Metrohm Tiamo software. For the pH adjustments, a Metrohm 781 pH meter paired with a Metrohm pH electrode was used. As a reference electrode was used, a Metrohm silver/silver (I) chloride electrode with a potassium chloride (3 M) electrolyte.

### 3.6. Procedure

For the described studies a prepared DHBI–TPB surfactant sensor paired with a reference electrode was used. The distilled water was employed for rinsing electrodes between measurements.

#### 3.6.1. Direct Potentiometric Sensor Characterization

The response measurements of DHBI–TPB surfactant sensor were performed by the incremental addition of cationic surfactants CTAB, CPC and Hyamine in deionized water. The concentrations of the cationic surfactants ranged from 5 × 10^−4^ M to 5 × 10^−5^ M, which corresponds to the logarithmic concentration area from approximately −2 to −8. The dynamic response measurements of DHBI–TPB surfactant sensor to TPB and DDS anions were performed using the activity step method according to IUPAC [30]. To reach certain concentrations, determined quantities of TPB and DDS anions were added to deionized water and gave a dynamic response, The speed of the response and sensor stabilization time were measured.

Interference measurements were performed by the incremental addition of CTAB in selected interfering cation solutions (0.01 M). The concentrations of CTAB were 5 × 10^−4^ M and 5 × 10^−5^ M to cover the logarithmic concentration area from approximately −2 to −8. The fixed interference method, as proposed by IUPAC [31], was used to determine the potentiometric selectivity coefficients.

The influence of the pH on the DHBI–TPB surfactant sensor response towards CTAB (0.5 mM) in deionized water and CTAB (0.5 mM) in Na_2_SO_4_ (0.1 M) was observed within the range of pH 2−13. Na_2_SO_4_ was used to observe the influence of the high ionic strength. The corresponding pH values were adjusted with HCl (0.5 M) and NaOH (0.5 M).

The deionized water was used for sensor storage. For the elemental analysis PerkinElmer 2400 CHNS/O Series II System was used (PerkinElmer Inc., Waltham, MA, USA).

#### 3.6.2. Direct Potentiometric Titrations

Direct potentiometric titrations were performed in the dynamic equivalent-point titration mode. The signal drift was set to 5 mV/min. Between the measurements, electrodes were rinsed with distilled water. The direct potentiometric titrations of cationic surfactants with DHBI–TPB surfactant sensor were measured in two directions; (a) to examine the influence of nonionic surfactants on CTAB titrations, and (b) to titrate cationic surfactants in commercial samples.

To check the influence of nonionic surfactants amount and number of EO-nonionic surfactant groups on the direct potentiometric titrations of mixtures containing CTAB and a selected nonionic surfactant, a series of three nonionic surfactants containing different numbers of EO groups was separately prepared and used in combination with CTAB and TPB as a titrant. The studied nonionic surfactants were Genapol T080 with 8 EO groups, Triton X100 with 10 EO groups and Genapol T110 with 11 EO groups, in two concentration series. These measurements were performed in 0.2 M Ba^2+^ solutions which was necessary to form a pseudoionic complex of Ba^2+^ with nonionic surfactants (3) that could be titrated with TPB solutions (buffered with borate buffer solution at pH 10.0).

The concentrations of cationic surfactants in commercial disinfectants and personal care products were measured with DHBI–TPB surfactant sensor by the direct potentiometric titrations of raw industrial cationic surfactant samples and 12 commercial samples––mouthwash, eye drops, nose drops, medical skin disinfectant, and hospital disinfectant––that were purchased from local drugstores.

The standard addition method was used to establish the matrix influence on end-point detection and for the determination of accuracy, precision, and matrix influence. A two-phase titration was used as an official reference method for the cationic surfactants. [12]. Three repetitive measurements were performed.

## 4. Conclusions

A new cationic surfactant DHBI-Br 1 was successfully synthesized and characterized by NMR, FTIR, LC-MS and elemental analysis. The exchange of a counter-ion of DHBI-Br 1 created the new ionophore DHBI–TPB 2, which was employed to develop the new PVC-based DHBI–TPB direct potentiometric surfactant sensor. This device presented excellent response characteristics to CTAB, CPC and Hyamine with a Nernstian slope from 57.1 to 59.1 mV/decade. The broadest useful linear concentration range was presented for CTAB, 2.0 × 10^−6^ to 1.0 × 10^−4^ M, with the lowest LOD of 0.3 × 10^−6^ M. Dynamic responses of DDS and TPB in the range of 1 × 10^−6^ to 1 × 10^−3^ M showed fast sensor response and a stable signal. Inorganic and organic cations that are usually found in commercial product formulations showed no significant influence on the DHBI–TPB surfactant sensor response towards CTAB. A pH from 2 to 11 did not influence the measurements in the range. Direct potentiometric titrations of cationic surfactants and nonionic surfactants in model formulations exhibited two inflexion points. The first was obtained because of the CTAB counter ion and the second because of to the pseudoionic complex of Ba^2+^ ions and EO-nonionic surfactant counter-ion. An increased number of EO groups (9 to 11) had an extensive negative influence on to the titration curve shape, inflexion point at the first equivalence point, and the potential change. The increased concentration of EO-nonionic surfactants showed a negative influence on titration curves at the first but also at the second inflexion point. The results of the direct potentiometric titrations of raw cationic surfactants––CTAB, Hyamine, CPC, BAC, DDAC––that were used in the production of disinfectants and personal care products, showed good agreement with a reference two-phase titration method. The standard addition method was successfully applied to commercial samples of mouthwash and eye drops containing cationic surfactants and showed recoveries from 98 to 102%. The results of direct potentiometric titrations of 12 commercially available disinfectants and personal care products containing cationic surfactants showed good agreement with the results obtained by the employment of a commercial surfactant sensor. As presented, the DHBI–TPB direct potentiometric surfactant sensor is cheap, simple, robust and user-friendly. Therefore, it presents an excellent new alternative for the routine analysis of cationic surfactants in commercial samples. Further studies on multicomponent mixtures containing cationic surfactants and nonionic surfactants are in progress.

## Figures and Tables

**Table 1:** Calculated response characteristics for the DHBI–TPB surfactant sensor to CTAB, CPC and Hyamine cationic surfactants with mean values at ±95% confidence limits.

Parameters	CTAB	CPC	Hyamine
Slope (mV/decade)	59.2 ± 0.5	58.4 ± 0.7	57.1 ± 1.1
Correlation coefficient (R^2^)	0.9999	0.9992	0.9988
Intercept (mV)	159.1 ± 7.0	199.6 ± 7.2	174.0 ± 5.1
Detection limit (M)	0.3 × 10^−6^	0.7 × 10^−6^	1.1 × 10^−6^
Useful linear concentration range (M)	2.0 × 10^−6^ to 1.0 × 10^−4^	3.0 × 10^−6^ to 1.0 × 10^−4^	3.0 × 10^−6^ to 1.0 × 10^−4^

**Table 2:** Calculated selectivity coefficient for different inorganic and organic anions mostly used in commercially formulated products measured with the DHBI–TPB surfactant sensor.

Interfering Cations	$K_{Cat_{i.}^{+}}^{pot}$
Na^+^	4.1 × 10^−5^
K^+^	2.5 × 10^−5^
NH_4_^+^	6.2 × 10^−5^
Mg^2+^	3.6 × 10^−5^
Ca^2+^	1.6 × 10^−5^
Zn^2+^	2.7 × 10^−5^
2-aminoethanol	0.3 × 10^−5^
Tris(2-hydroxyethyl)amine	1.1 × 10^−5^
Tetraethylazanium	2.8 × 10^−5^
Triton B	1.5 × 10^−5^
Benzyltriethylammonium	5.2 × 10^−5^

**Table 3:** The direct potentiometric titrations of technical-grade cationic surfactants by the DHBI–TPB surfactant sensor compared to a standard two-phase titration method. For direct potentiometric titrations, TPB (4 × 10^−3^ M) was used as a titrant.

Technical Grade Cationic Surfactant	DHBI–TPB Surfactant Sensor		Two-Phase Titration
	Found (%)	RSD (%)	Found (%)
CTAB	95.39	0.59	95.89
Hyamine	98.45	0.48	98.52
CPC	97.92	0.34	98.20
BAC	51.09	0.90	50.86
DDAC	50.97	0.61	51.43

**Table 4:** The results of the direct potentiometric titrations of the commercial samples with the standard addition method by adding the known quantity of CTAB and by using the DHBI–TPB surfactant sensor as the end-point indicator and DDS (4 × 10^−4^ M) as the titrant.

Commercial Sample	Calculated Cationic Surfactant (M)	CTAB		
		Added (mol)	Found (mol) ^a^	Recovery (%)
Mouthwash 1	3.34 × 10^−3^	1 × 10^−5^	0.98 × 10^−5^	98.0
Mouthwash 2	3.83 × 10^−3^	7 × 10^−5^	7.11 × 10^−5^	101.6
Mouthwash 3	1.40 × 10^−3^	3 × 10^−4^	3.06 × 10^−5^	102.0
Eye drops 1	6.26 × 10^−4^	5 × 10^−5^	5.03 × 10^−5^	100.6

^a^ Average of three measurements.

**Table 5:** The results of direct potentiometric titrations of twelve commercially available disinfectants and personal care products containing cationic surfactants using the TPB (5 × 10^−3^ M) as a titrant and DHBI–TPB surfactant sensor as the end-point indicator. The results were compared with the results obtained from the commercial surfactant sensor and a referent two-phase titration method.

Type Of Commercial Product	DHBI–TPB Surfactant Sensor ^a^			Commercial Surfactant Sensor ^b^		Two Phase Titration ^b^
	Found%	RSD(%)	Confidence Interval (±)	Found%	RSD(%)	Found%
Mouthwash 1	0.1137	0.74	0.000743	0.1151	0.76	0.1143
Mouthwash 2	0.1303	1.06	0.001216	0.1297	1.12	0.1311
Mouthwash 3	0.0480	1.49	0.000630	0.0480	1.39	0.0462
Mouthwash 4	0.1882	0.34	0.000560	0.1888	0.39	0.1821
Medical skin disinfectant 1	0.1998	1.27	0.002231	0.1984	1.32	0.1993
Medical skin disinfectant 2	0.3769	0.91	0.000788	0.3639	0.89	0.3649
Disinfectant for hospitals 1	4.1032	0.97	0.001862	4.1005	0.91	4.1025
Disinfectant for hospitals 2	5.0964	0.51	0.000982	5.0979	0.78	5.1002
Nose drops 1	0.0150	1.76	0.000232	0.0152	1.06	0.0142
Nose drops 2	0.0151	2.07	0.000275	0.0160	1.90	0.0156
Nose drops 3	0.0099	1.49	0.000129	0.0102	1.67	0.0101
Nose drops 4	0.0178	1.67	0.000261	0.0185	1.73	0.0180

^a^ Average of five measurements. ^b^ Average of three measurements.

## Data Availability

The data presented in this study are available in Supplementary Materials.

## Supplementary Materials

The following are available online, Figures S1 to S4: titles as indicated in Table of Contents.

## Sample Availability

All samples are available from the authors.
