# Supplementary material for: Direct Potentiometric Study of Cationic and Nonionic Surfactants in Disinfectants and Personal Care Products by New Surfactant Sensor Based on 1,3-Dihexadecyl−1H-benzo[d]imidazol−3-ium
Source: Molecules. 2021 Mar 4;26(5):1366. doi: 10.3390/molecules26051366 (PMC7961908; doi:10.3390/molecules26051366)
Supplement: Supplementary file 1 [file molecules-26-01366-s001.pdf]

Direct Potentiometric Study of Cationic and Nonionic Surfactants in Disinfectants and Personal Care Products by New Surfactant Sensor Based on 1,3-Dihexadecyl-1*H*-benzo[*d*]imidazol-3-ium

Nikola Sakač[1*], Dean Marković[2], Bojan Šarkanj[3], Dubravka Madunić-Čačić[1,4], Krunoslav Hajdek[5], Božo Smoljan[5], Marija Jozanović[6*]

[1]Faculty of Geotechnical Engineering, University of Zagreb, Hallerova 7, 42000 Varaždin, Croatia
[2]University of Rijeka, Department of Biotechnology, Radmile Matejčić 2, 51000 Rijeka, Croatia
[3]University North, Department of Food Technology, Trg dr. Žarka Dolinara 1, 48000 Koprivnica, Croatia
[4]Saponia Chemical, Pharmaceutical and Foodstuff Industry, Inc., M. Gupca 2, 31000 Osijek, Croatia
[5]University North, Department of Packaging, Recycling and Environmental protection, Trg dr. Žarka Dolinara 1, 48000 Koprivnica, Croatia
[6]Department of Chemistry, University of Osijek, Cara Hadrijana 8A, HR-31000 Osijek, Croatia

*** Correspondence:**

nikola.sakac@gfv.unizg.hr; Tel.: +385915830336; Fax.: +385 (0)42313587

mjozanovic@kemija.unios.hr; Tel.: +385996865716; Fax.: +38531399969

Table of contents

1. FTIR spectrum of powder 1,3-dihexadecyl-1*H*-benzo[*d*]imidazol-3-ium (**1**). S1

2. ^{1}H NMR of 1,3-dihexadecyl-1*H*-benzo[*d*]imidazol-3-ium bromide (**1**) S2

3. ^{13}C APT NMR of 1,3-dihexadecyl-1*H*-benzo[*d*]imidazol-3-ium bromide (**1**). S3

4. Positive ESI-MS/MS Q1 scan for 1,3-dihexadecyl-1*H*-benzo[*d*]imidazol-3-ium (**1**); infusion 10 μL min^{-1} at concentration 2.5 ng μL^{-1}; S4

1,3-Dihexadecyl-1*H*-benzo[*d*]imidazol-3-ium bromide (1)

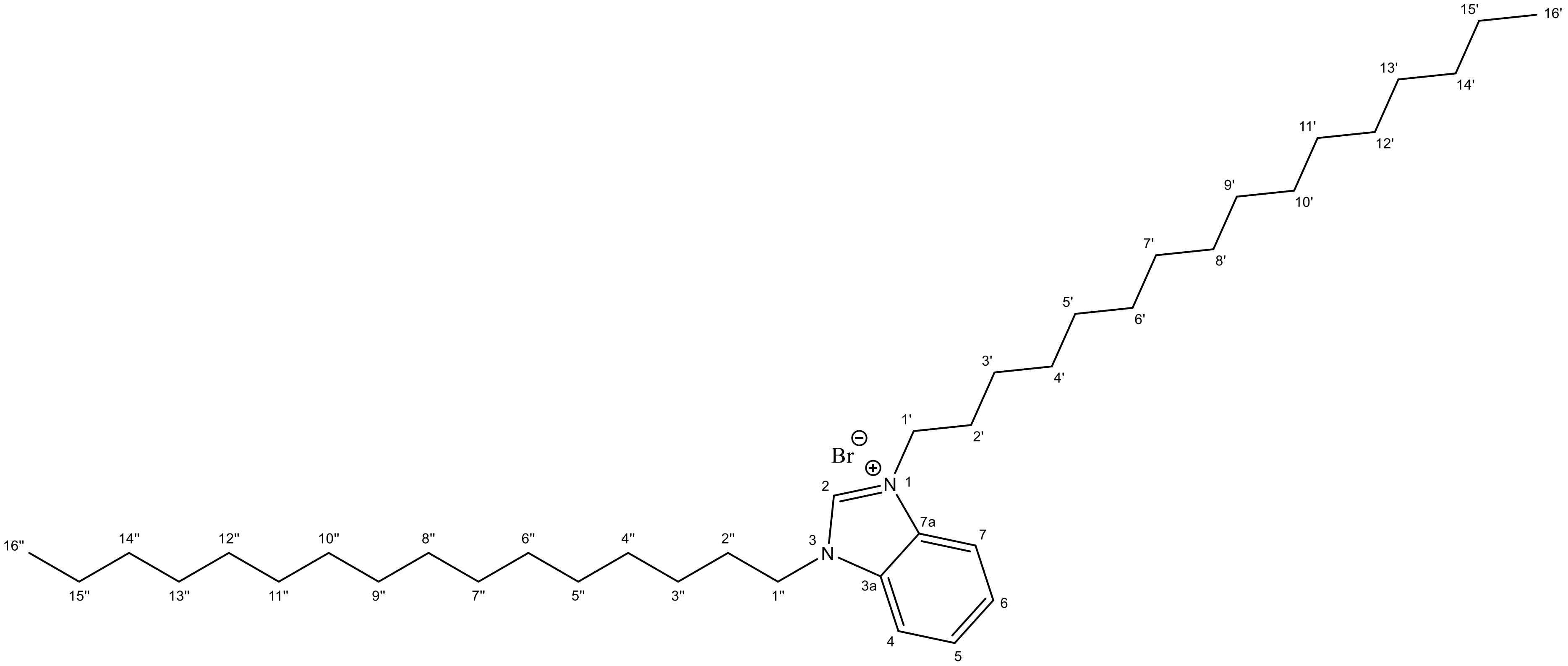

The alkylation reaction of benzimidazol (0.312 g, 2.64 mmol) was performed under basic conditions by addition of $NaHCO_3$ (0.887 g, 10.56 mmol, 4 equiv.) in anhydrous dimethylformamide (15 mL) and an excess of 1-bromohexadecane (4.84 g, 15.84 mmol, 6 equiv.). The progress of the reaction was followed by TLC (DCM/methanole = 10:0.25) and the reaction was carried out under reflux for 48h in an inert nitrogen atmosphere. NaBr salt was precipitated with hexane and the crude product **1** was obtained by filtration. Further purrification by flash chromatography (DCM/methanole = 10:0.25) gave the desired bisalkylated QAC **1** (0.941 g, 1.66 mmol) in 62.7% yield.

White powder, 62,7% yield (0.941 g, 1.66 mmol); m.p.= 96.3-99.2 ℃; R_f = 0.47 (DCM/methanole = 10:0.25); **^{1}H NMR** ($CDCl_3$, 400 MHz): δ = 11.50 (s, 1H, H-C(2)), 7.75–7.62 (m, 4H, H-C(4), H-C(5), H-C(6), H-C(7)), 4.63 (t, J= 7.3, 4H, H-(C1'), H-(C1")), 2.05 (tt, J= 7.4, 7.3, 4H, H-

(C2'), H-(C2")), 1.41 (tt, J= 7.3, 7.3, 4H, H-(C3'), H-(C3")), 1.34 (tt, J= 7.3, 7.3, 4H, H-(C4'), H-(C4")), 1.31–1.17 (m, 44H, H-(C5')-H-(C15'), H-(C5")- H-(C15")), 0.87 (t, J= 7.4, 6H, H-(C16'), H-(C16")). **^{13}C NMR** ($CDCl_3$, 100.6 MHz): δ = 142.98 (2C), 131.34 (1C), 127.05 (2C), 113.05 (2C), 47.68 (2C), 31.91 (2C), 29.69 (2C), 29.68 (2C), 29.66 (2C), 29.65 (2C), 29.63 (2C), 29. 58 (2C), 29.55 (2C), 29.49 (2C), 29.38 (2C), 29.35 (2C), 29.04 (2C), 26.56 (2C), 22.68 (2C), 14.11 (2C). **IR** (KBr, cm^{-1}): 3480, 3405, 3115, 3035, 3030, 2955, 2920, 2850, 2035, 1990, 1950, 1910, 1810, 1790, 1720, 1610, 1460, 1425, 1380, 1350, 1300, 1280, 1215, 1200, 1190, 1140, 1125, 1110, 1070, 1015, 880, 845. **MS-CI** (NH_3): 568, 536, 445, 381, 371, 353, 338, 304. **Elem. anal.** (calc. for $C_{39}H_{71}BrN_2$): C 72.30; H 11.05; N 4.32; found: C 72.25; H 11.02; N 4.27.

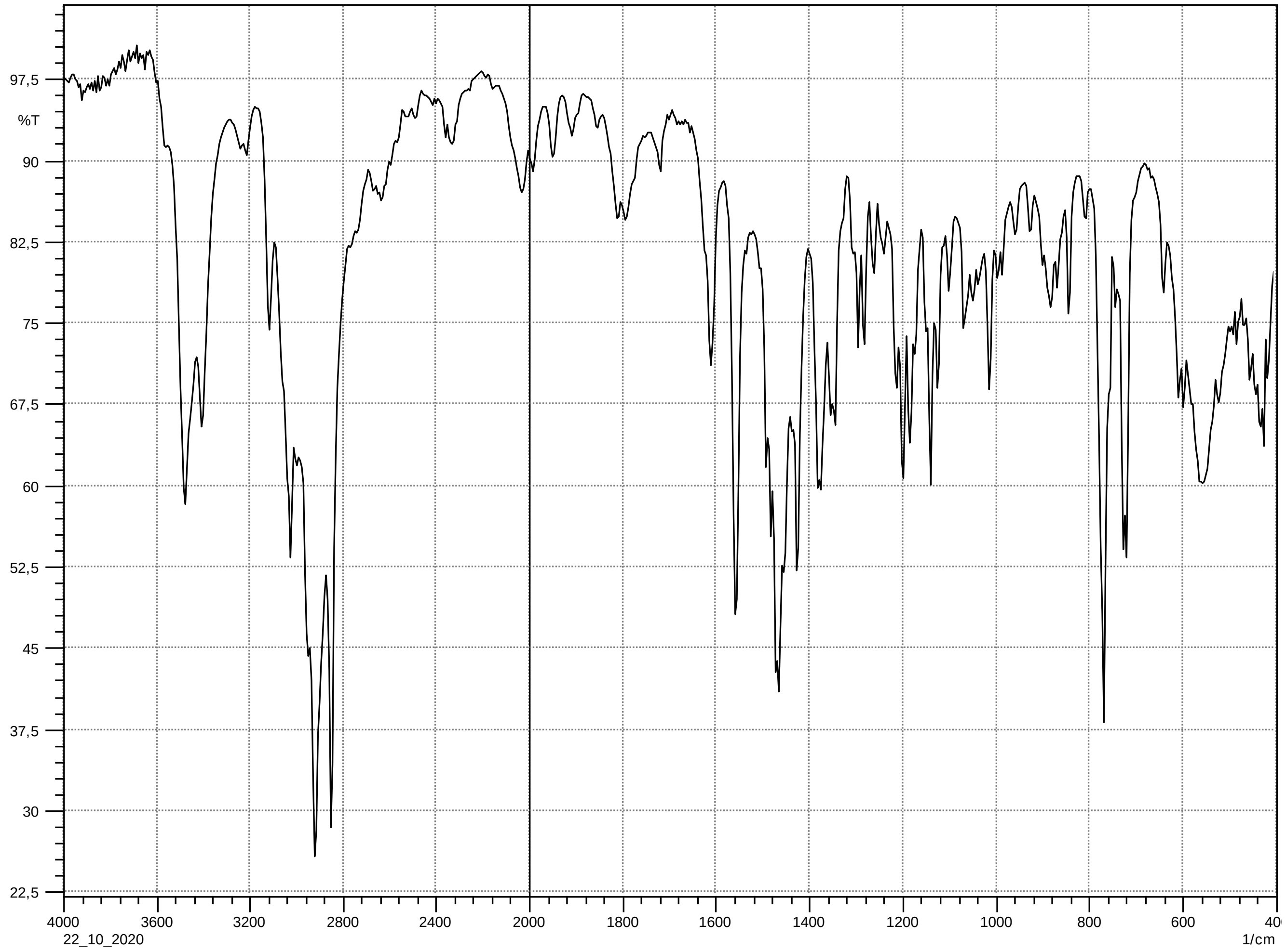

Figure S1. FTIR spectrum of powder 1,3-dihexadecyl-1*H*-benzo[*d*]imidazol-3-ium.

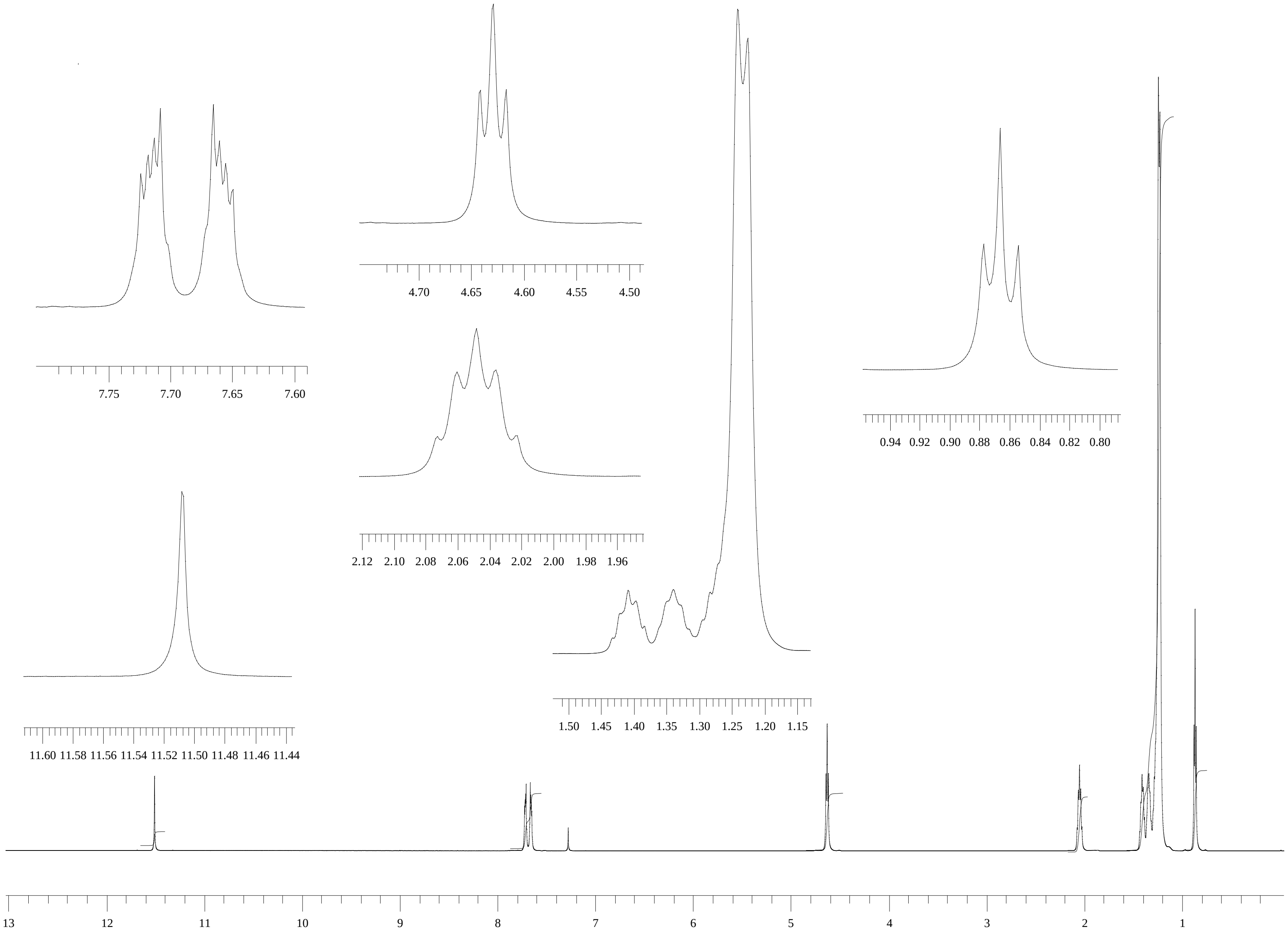

Figure S2. ^{1}H NMR of 1,3-dihexadecyl-1*H*-benzo[*d*]imidazol-3-ium bromide (**1**).

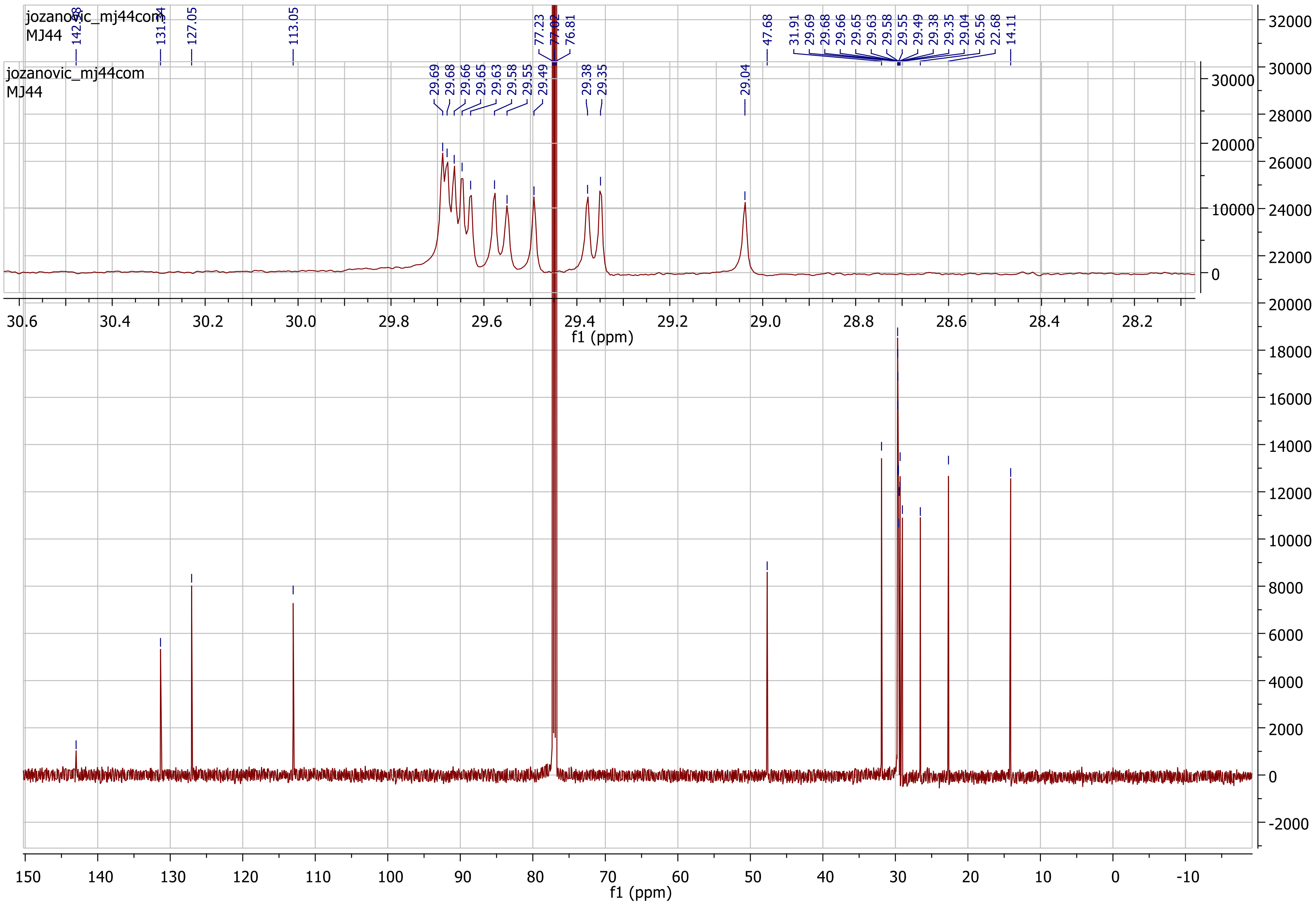

Figure S3. ^{13}C NMR of 1,3-dihexadecyl-1*H*-benzo[*d*]imidazol-3-ium bromide (**1**).

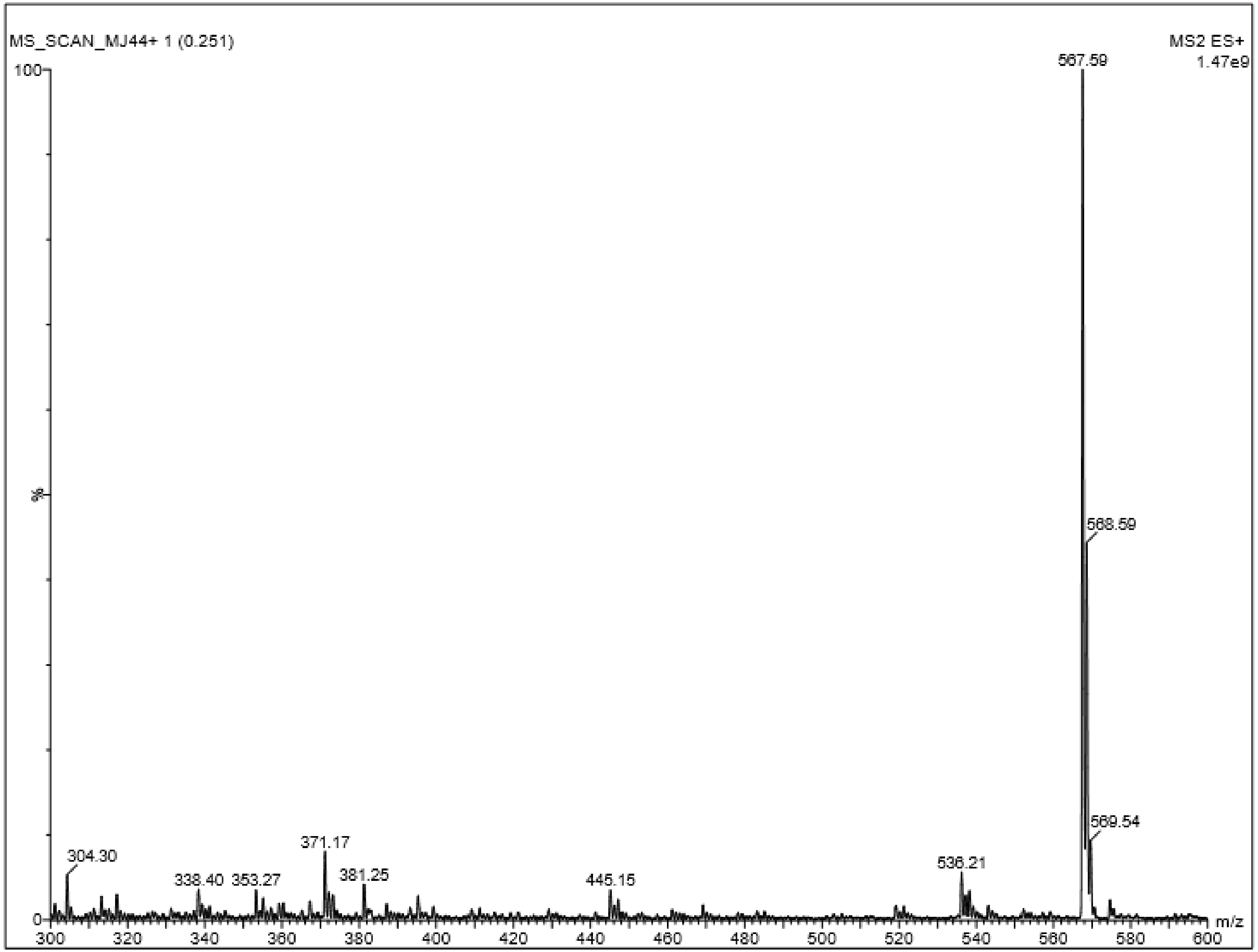

Figure S4. Positive ESI-MS/MS Q1 scan for 1,3-dihexadecyl-1*H*-benzo[*d*]imidazol-3-ium; infusion 10 μL min^{-1} at concentration 2.5 ng μL^{-1}.
